# OP12 Conceptual Care Pathway And Cost-Consequence Analysis Of Telehealth In Care Delivery To Neurodivergent Children And Young People

**DOI:** 10.1017/S0266462325100718

**Published:** 2025-12-29

**Authors:** Yanga Nokhepheyi, Jason Lang, Kathleen Boyd

## Abstract

**Introduction:**

Neurodivergent children and young people (CYP) experience delays in accessing diagnostic services (two years and three months in the UK). This may harm their development and create missed opportunities for support. Telehealth offers alternative solutions to streamline services and improve the identification of neurodivergence. An early economic analysis was developed to estimate telehealth’s likely costs and consequences in delivering care to CYP.

**Methods:**

A conceptual care pathway involving assessment using a telehealth platform—vCreate Neuro—compared to an assessment without vCreate Neuro, was derived from expert consultation and published UK clinical guidelines. A cost-consequence analysis was conducted from a societal perspective in Glasgow, Scotland using data from ongoing pilot studies in National Health Service (NHS) and published literature. Surveys and expert consultations with clinicians assessed trade-offs between costs and consequences associated with the delivery of care in the care pathway. Cost data included direct and overhead expenses related to service delivery.

**Results:**

Costs incurred in the vCreate Neuro pathway were estimated to be GBP1,141.50 (USD1,569.50) and GBP1,220.00 (USD1,677.45) (per patient) without vCreate Neuro, which resulted in GBP78.50 (USD107.90) savings. The clinician survey results indicated possible improvements in time to diagnosis by several weeks to months, with an average decrease of up to four unnecessary appointments. Clinician-to-patient relationships and quality of care may also improve, with 90 minutes of medical time liberated per patient. Additionally, clinicians reported potential improvements in the quality of relationships with parents. Results from the expert consultation highlighted a likely increase in administrative burden for clinicians using the telehealth platform.

**Conclusions:**

The possible administrative burden of using the vCreate Neuro care pathway was rewarded by resource use savings and reduced time to diagnosis. Further evaluation of the system’s costs, benefits, and acceptability by parents or carers is required. This study is relevant for health systems with limited resources, complex services requiring multidisciplinary teams, and the need to overcome barriers to care.

